# Determination and prediction of standardized ileal amino acid digestibility of wheat shorts for pullets during the brooding and growing phases

**DOI:** 10.5713/ab.260107

**Published:** 2026-06-15

**Authors:** Chong Chen, Shiqian Liu, Kaiyue Ren, Liang Zhao, Tong Xing, Liren Ding, Feng Gao, Lin Zhang

**Affiliations:** 1 College of Animal Science and Technology, Key Laboratory of Animal Origin Food Production and Safety Guarantee of Jiangsu Province, Jiangsu Collaborative Innovation Center of Meat Production and Processing, Quality and Safety Control, Nanjing Agricultural University, Nanjing, China

**Keywords:** Amino Acids, Prediction Equations, Pullet, Standardized Ileal Digestibility, Wheat Shorts

## Abstract

**Objective:**

This study was conducted to evaluate the nutritional value of ten wheat shorts (WS) originating from different regions by determining their standardized ileal digestibility of amino acids (SID AA) in pullets during the brooding (d 28–31) and growing (d 92–95) phases, and to establish SID AA prediction equations based on their chemical composition.

**Methods:**

A total of 990 pullets were used, with 594 in the brooding phase and 396 in the growing phase. In each phase, birds were randomly assigned to one of 11 dietary treatments, including one nitrogen-free diet and ten test diets each formulated with an individual WS sample as the sole protein source. All diets contained 0.5% titanium dioxide as an indigestible marker.

**Results:**

Substantial variation (CV>10%) was observed in ether extract, crude ash, crude fiber, acid detergent fiber (ADF), neutral detergent fiber, and total starch (TS) among the WS samples. During the brooding phase, SID was lowest for cysteine (74.10%) and highest for glutamic acid (90.06%). During the growing phase, cysteine was again lowest (70.33%) and glutamic acid highest (90.22%). Except for tryptophan, the WS source significantly affected SID AA (p<0.05). In the brooding phase, ADF, TS, and gross energy (GE) were identified as significant predictors of SID AA. Similarly, ADF and GE were reliable predictors in the growing phase.

**Conclusion:**

Considerable variation was observed in the chemical composition of WS from different origins, and the origin had a significant effect on SID AA. During the brooding phase, the equation with the highest R^2^ was SID Met = 51.929+1.015TS− 0.494NDF (R^2^ = 0.976; p<0.001). During the growing phase, the equation with the highest R^2^ was SID His = −511.489−2.095ADF+23.722GE+1.652NFE (R^2^ = 0.900; p<0.001).

## INTRODUCTION

Wheat shorts (WS), as one of the important by-products of wheat milling, are mainly composed of wheat germ, the aleurone layer, pericarp, and part of the endosperm, with crude fiber (CF) content ranging from 4% to 7% [1]. Owing to its balanced nutritional composition and low cost, WS are widely used as a feed ingredient in livestock and poultry diets [2]. However, differences among wheat varieties, growing environments, and processing technologies often lead to significant fluctuations in the chemical composition of WS [3,4], which limits its application to some extent. Moreover, although many studies have reported the standardized ileal digestibility of amino acids (SID AA) of feed ingredients in poultry [5,6], most studies have focused on broilers, while data on the digestibility of WS in pullets remain very limited. Therefore, to enable the precise application of WS in pullet feed formulation, it is necessary to determine the amino acid digestibility of WS in pullets.

SID AA is a widely adopted evaluation index in livestock and poultry feed formulation because it is derived from apparent ileal digestibility of amino acids (AID AA) by correcting for basal endogenous amino acid losses [7]. Thus, unlike apparent ileal digestibility values, SID AA values are not influenced by dietary amino acid concentrations and exhibit additivity among feed ingredients in mixed diets, thereby facilitating more precise feed formulation [8,9]. Furthermore, prediction equations established based on the correlations between feed chemical composition (including conventional nutrients and amino acid profiles) and SID AA allow for rapid and economical estimation of amino acid digestibility, thereby reducing reliance on animal trials. Such prediction approaches have been applied in studies on various feed ingredients. For example, Liu et al. [6] successfully established prediction equations for the SID AA of wheat bran in broilers at 13 and 28 days of age based on its chemical composition, with crude protein (CP), CF, acid detergent fiber (ADF), neutral detergent fiber (NDF), and crude ash (ash) serving as the key predictors. However, for WS, although previous studies have determined the SID AA and developed corresponding prediction equations in pigs [10], comparable studies on WS in pullets have not yet been conducted. Moreover, given that the digestibility of the same feed ingredient can vary across physiological stages [11], conducting feeding trials with WS specifically in pullets at different growth phases is particularly necessary. Accordingly, this study was designed to collect 10 WS samples from major producing regions across China, systematically analyze the variations in their chemical composition, and determine their SID AA in pullets during the brooding and growing phases. Subsequently, prediction equations for SID AA were established based on the conventional nutrient composition and amino acid profiles of WS, thereby providing data support and a predictive tool for formulating pullet diets using WS.

## MATERIALS AND METHODS

### Wheat shorts sampling

The ten WS samples (coded WS1 to WS10) were collected from the following locations across China: Weinan (Shaanxi Province), Weifang (Shandong Province), Chengdu (Sichuan Province), Xingtai (Hebei Province), Linfen (Shanxi Province), Chengdu (Sichuan Province), Nanjing (Jiangsu Province), Huainan (Anhui Province), Zhumadian (Henan Province), and Beijing. All samples were ground and sieved through a 40-mesh screen to obtain a more uniform particle size and improve sample homogeneity, thereby facilitating subsequent chemical analyses. The samples were stored at 4°C to minimize moisture absorption and potential nutrient deterioration before analysis.

### Animals, experimental design and sample procedure

This study was conducted in two phases with Jingfen No. 8 (a commercial laying hen breed, Beijing Huadu Yukou Poultry Industry): the brooding phase (days 28–31) and the growing phase (days 92–95). In each phase, birds were randomly assigned to one of 11 dietary treatments. The brooding phase comprised 594 pullets, with nine birds per cage and six replicate cages per treatment. The growing phase comprised 396 pullets, with six birds per cage and six replicate cages per treatment. During the entire experimental period, pullets were kept in three-tiered stacked cages and had free access to feed and water. The daily management included feeding at 07:00, 11:00, and 18:00, and a constant 12-hour lighting schedule was maintained. Temperature began at 33°C and was lowered by 3°C per week until reaching a final level of 24°C. Relative humidity was controlled at 65%–70% in the first week and then adjusted to 50%–60%. The experimental diets consisted of one nitrogen-free diet and ten test diets formulated with WS as the major component. Titanium dioxide (TiO_2_) was added at 0.5% to all diets as an index. The detailed ingredient composition and nutrient levels of the experimental diets are presented in Tables 1, 2.

At 31 or 95 days of age, the pullets were humanely euthanized by CO_2_ asphyxiation in a stunning chamber, followed by exsanguination via the carotid artery. The abdominal cavity was opened, and the entire ileum was rapidly isolated. Then, the contents of the posterior ileum (excluding the terminal 2 cm) were gently flushed into sampling containers using distilled water. Immediately after collection, ileal digesta collected from birds within the same cage were pooled as one sample. In total, 594 pullets in the brooding phase and 396 pullets in the growing phase were used for sampling. The pooled digesta samples were snap-frozen and subsequently freeze-dried. The freeze-dried samples were stored at −20°C until analysis.

### Measurements

For WS samples and experimental diets, dry matter (DM), CP, crude ash (ash), calcium (Ca), total phosphorus (TP), and ether extract (EE) were determined according to AOAC [12] methods (930.15, 984.13, 942.05, 968.08, 964.06 and 920.39, respectively). NDF and ADF were determined using an ANKOM A200i fiber analyzer (Ankom Technology) with fiber filter bags, according to the procedures modified from Van Soest et al. [13]. For NDF, samples were treated with neutral detergent solution containing heat-stable α-amylase and sodium sulfite to obtain the cell wall residue. For ADF, samples were treated with acid detergent solution containing cetyltrimethylammonium bromide to obtain the cellulose and lignin residue. CF was determined using the same analyzer with fiber filter bags, in which samples were sequentially digested with dilute acid and dilute alkali, and the remaining organic residue was quantified after ashing. Nitrogen-free extract (NFE) was calculated on an as-fed basis as: NFE (%) = 100−(Moisture [%]+CP [%]+EE [%]+CF [%]+Ash [%]). Gross energy (GE) was measured using an oxygen bomb calorimeter (Model TYHW-V; Henan Tianyu Instrument Manufacturing). Total starch (TS) content was determined using a Solarbio starch assay kit (Catalog No.: BC0705-100T/96S). Phytic acid (PA) content was determined using a Solarbio PA assay kit (Catalog No.: BC5840-50T/24S). The analytical procedures were performed strictly according to the manufacturers’ instructions without modification. Both kits were validated for use with feed ingredients, and preliminary experiments were conducted prior to formal analysis to confirm the applicability of the methods.

For WS, experimental diets, and digesta samples, tryptophan was analyzed by alkaline hydrolysis: samples were placed in polytetrafluoroethylene tubes, hydrolyzed with lithium hydroxide at 110°C for 20 h, diluted to volume, filtered, and then measured using a liquid chromatography system (U3000; Thermo Fisher Scientific). The other 17 amino acids were determined after pretreatment according to AOAC [14] using an automatic amino acid analyzer (LA8080, Hitachi). Specifically, for sulfur-containing amino acids (cysteine and methionine), samples were pre-oxidized with performic acid for 16 h, and the reaction was terminated with sodium metabisulfite. Samples were then hydrolyzed with HCl at 110°C for 24 h. The hydrolysate was filtered, made up to volume, and analyzed using the same analyzer. The remaining 15 amino acids were determined by acid hydrolysis with HCl in sealed ampoules at 110°C for 24 h. The hydrolysate was filtered, made up to volume, and analyzed using the same analyzer. Titanium dioxide (TiO_2_) content in diets and digesta was determined according to the method described by Myers et al. [15].

### Calculations

In this study, the AID AA, SID AA, and ileal endogenous amino acid (IEAA) were calculated using the formula described by Cao et al. [16], with all data expressed on a DM basis:


(1) $$\text{IEAA }(\text{mg}/\text{kg})=\text{Amino }{\text{acid}}_{\text{ileal}}\times ({\text{T}}_{\text{diet}}/{\text{T}}_{\text{ilea}})$$

Where amino acid_ileal_ represents the concentration of amino acids in ileal digesta, T_diet_ denotes the marker concentration in the diet, and T_ileal_ indicates the marker concentration in ileal digesta.


(2) $$\text{AID AA }(\%)=(1-[{\text{T}}_{\text{diet}}/{\text{T}}_{\text{ileal}}]\times [\text{Aimno }{\text{acid}}_{\text{ileal}}/\text{Aimno }{\text{acid}}_{\text{diet}}])\times 100$$

Where amino acid_diet_ represents the concentration of amino acids in the diet.


(3) $$\text{SID AA}(\%)=\text{AID AA}+(\text{IEAA}/\text{Aimno }{\text{acid}}_{\text{diet}})\times 100$$

### Data statistics and analysis

Experimental data were initially organized using Microsoft Excel 2019. All data were analyzed using SPSS Statistics 27.0 (IBM). One-way analysis of variance (ANOVA) was performed to compare SID AA values among the ten WS sources, and Duncan’s multiple range test was applied for post hoc pairwise comparisons. Pearson correlation analysis was conducted to examine the relationships between the chemical composition of WS and SID AA. Stepwise multiple linear regression was applied to develop prediction equations for SID AA based on the chemical composition of WS. Differences were considered statistically significant at p<0.05 and highly significant at p<0.01. The coefficient of determination (R^2^) was used to indicate the goodness of fit for the prediction equations.

## RESULTS

### Chemical properties of wheat shorts

The chemical composition and amino acid content of the WS samples are shown in Table 3. On a DM basis, the average contents were as follows: DM 89.05% (87.43% to 90.35%), CP 17.62% (15.71% to 19.51%), EE 4.00% (2.54% to 5.27%), ash 3.66% (2.52% to 4.91%), CF 5.04% (2.14% to 10.67%), NFE 69.68% (64.75% to 75.69%), NDF 32.38% (25.02% to 43.28%), ADF 7.51% (4.12% to 14.47%), TS 42.20% (37.06% to 51.58%), Ca 0.09% (0.07% to 0.12%), TP 1.00% (0.70% to 1.34%), PA 2.16% (1.88% to 2.44%), and GE 20.73 MJ/kg (20.32 to 21.17 MJ/kg). TS, EE, ash, CF, NDF, ADF, Ca, and TP exhibited high variation (CV>10%), with CF showing the highest CV (44.28%). Amino acid analysis showed an average lysine content of 0.74% (0.55% to 0.88%), methionine 0.29% (0.25% to 0.36%), tryptophan 0.20% (0.16% to 0.23%), and threonine 0.56% (0.48% to 0.63%). The total amino acid (TAA) content averaged 15.07% (13.08% to 16.71%; CV = 7.39%), the essential amino acid (EAA) content averaged 6.32% (5.35% to 7.06%; CV = 9.02%), and the non-essential amino acid (NEAA) content averaged 8.76% (7.74% to 9.65%; CV = 6.75%).

### Ileal endogenous amino acid losses in the pullets

The IEAA flows of all amino acids are presented in Table 4. For 31-d-old pullets, the EAA flows ranged from 147.82 mg/kg (tryptophan) to 767.61 mg/kg (leucine), while NEAA flows ranged from 324.93 mg/kg (cysteine) to 1,293.90 mg/kg (glutamic acid). For 95-d-old pullets, the EAA flows ranged from 130.04 mg/kg (tryptophan) to 1,149.28 mg/kg (leucine), while the NEAA flows ranged from 405.08 mg/kg (cysteine) to 1,954.73 mg/kg (glutamic acid).

### Standard ileal amino acid digestibility

The SID AA of WS samples in pullets during the brooding phase and growing phase were presented in Tables 5, 6, respectively. During the brooding phase, differences were observed (p<0.05) in the SID AA of the ten WS samples for all AA except tryptophan. Among the EAA, the lowest SID was recorded for threonine (77.49%), while the highest was observed for arginine (86.66%). For the NEAA, cysteine exhibited the lowest SID (74.10%), and glutamic acid showed the highest (90.06%). At the individual source level, WS9 consistently yielded the highest SID values across most amino acids, whereas WS1 and WS2 generally showed the lowest values. In the growing phase, the SID AA values of the ten WS from different sources also differed (p<0.05). Among the EAA, the SID ranged from the lowest for threonine (74.48%) to the highest for arginine (88.75%). Regarding the NEAA, the lowest SID was found for cysteine (70.33%), and the highest was observed for glutamic acid (90.22%). WS5 and WS9 generally exhibited relatively higher SID AA values, while WS1 tended to show lower values. A comparison of the SID AA of WS between 31-day-old and 95-day-old pullets is shown in Table 7, where no differences were detected between the two phases.

### Correlation analysis

During the brooding phase, with the exception of tryptophan, the SID of the remaining amino acids was negatively correlated with ash, CF, and ADF, and positively correlated with TS (p<0.05; Supplement 1). GE and EE were negatively correlated with the SID of phenylalanine, threonine, valine, isoleucine, leucine, serine, glutamic acid, and proline (p<0.05). Other than aspartic acid, lysine, and tryptophan, the SID of the other amino acids was negatively correlated with PA (p<0.05) and positively correlated with NFE (p<0.05). With the exception of arginine, lysine, tyrosine, and tryptophan, the SID of the remaining amino acids was negatively correlated with the contents of arginine and lysine (p<0.05; Supplement 2). The SID of arginine, lysine, methionine, leucine, phenylalanine, isoleucine, histidine, and alanine was positively correlated with proline (p<0.05).

During the growing phase, the SID of all amino acids, except for lysine, alanine, and tryptophan, was negatively correlated with ADF (p<0.05; Supplement 3). Ash was negatively correlated with the SID of methionine, phenylalanine, arginine, serine, glutamic acid, proline, cysteine, and tyrosine (p<0.05). CF was negatively correlated with the SID of phenylalanine, histidine, arginine, threonine, serine, glutamic acid, proline, and tyrosine (p<0.05). The PA was negatively correlated with the SID of methionine, glutamic acid, and proline (p<0.05). Furthermore, the SID of methionine was also negatively correlated with the contents of arginine, valine, glycine, and alanine (p<0.05; Supplement 4). The SID of proline was negatively correlated with the contents of arginine, threonine, aspartic acid, glycine, and alanine (p<0.05). The SID of cysteine was negatively correlated with the contents of lysine, valine, arginine, threonine, aspartic acid, glycine, and alanine (p<0.05).

### Prediction equations for standardized ileal digestibility of amino acids

Correlation analysis between the conventional nutrients, anti-nutritional factors of WS, and their amino acid digestibility was conducted, leading to the development of prediction equations (Table 8). For the brooding phase, 18 SID AA prediction equations were established for WS. Most equations used TS, ADF, and GE as predictors, with the equation for SID Methionine demonstrating the best fit: SID Methionine = 51.929+1.015TS−0.494NDF (R^2^ = 0.976). Similarly, 16 SID AA prediction equations were developed for the growing phase. Most equations utilized ADF and GE as predictors. The equation with the highest coefficient of determination was for SID Histidine: SID Histidine = −511.489−2.095ADF+ 23. 722GE+1.652NFE (R^2^ = 0.900).

## DISCUSSION

In this study, ten WS samples from major wheat-producing regions in China were collected for chemical composition analysis. The results revealed a certain degree of variation (CV>10%) in core components, including CF, NDF, ADF, EE, ash, Ca, TS, and TP, reflecting the compositional diversity of samples collected from different regions. Among these, CF exhibited the highest CV (44.28%), and the CF content of WS1 was numerically higher than that of the other groups, likely due to the retention of an excessive amount of bran during its production. Notably, the variability observed was consistent with the high fluctuations (CV>10%) in EE, NDF, ADF, ash, Ca, TP, and TS reported by Huang et al. [10], and the contents of CP, ADF, and EE were within the ranges they reported. Specifically, while the average Ca (0.09%) and TP (1.00%) contents were consistent with NRC [17], the NDF (25.02% to 43.28%) and ADF (4.12% to 14.47%) values encompassed those reported by Huang et al. [10] and Villamide et al. [18]. The DM (89%) and CF (5.47%) were closely aligned with Huang et al. [10]. The mean TS (42.20%) and GE (20.73 MJ/kg) contents were significantly higher than those in previous reports [19,20], though similar to the findings of Huang et al. [10]. These discrepancies were attributed to differences in wheat growing environments, processing methods, and the regulatory effect of the fiber-starch ratio on energy value. Regarding amino acid composition, glutamic acid was the most abundant at 3.38% (3.04% to 3.78%) and tryptophan the least abundant at 0.20% (0.16% to 0.23%) in the WS analyzed, a pattern consistent with the findings of Stas et al. [20]. Overall, the amino acid contents determined in this study aligned with those reported by Villamide et al. [18].

The IEAA losses measured in the present study were higher than those previously reported for 12-wk-old, 30-wk-old, and 50-wk-old laying hens [16,21,22]. This could be partly attributed to differences in the composition of the nitrogen-free diet, as the ratio of glucose to corn starch and the amylose-to-amylopectin ratio have been shown to influence endogenous amino acid flows [23,24]. Numerically, IEAA losses at 95 d were generally higher than those at 31 d for most amino acids, a trend also observed by Kang et al. [25] in older laying hens. This may be attributable to enhanced intestinal mucosal turnover and increased enzyme secretion with advancing age in laying hens [26]. Among the IEAA losses measured in 31-d-old and 95-d-old pullets in this study, glutamic loss was the highest, followed by aspartic acid. It has been suggested that the predominance of glutamic acid in ileal endogenous flow is primarily attributable to the amino acid composition of mucoproteins, the major source of endogenous gut protein, which are particularly rich in glutamic acid, aspartic acid, serine, threonine, and proline [27,28]. Furthermore, when a nitrogen-free diet is fed, the absence of dietary nitrogen triggers the mobilization of glutamine from body protein reserves and its subsequent conversion to glutamic acid in the intestine [29], further contributing to the high glutamic acid flow.

In this study, the lowest SID among amino acids in WS for pullets was for cysteine, a result consistent with previous reports [30]. Furthermore, comparisons of the ten WS sources revealed that samples with higher NDF content had lower SID AA values in pullets, a finding supported by Adedokun et al. [31]. Adedokun et al. [31] suggested that the high NDF components may increase digesta viscosity, thereby hindering the efficient digestion and absorption of amino acids. Additionally, the physical abrasion of fiber on the intestinal epithelium may stimulate greater endogenous amino acid losses, further affecting amino acid digestibility. Significant differences (p<0.05) were observed in the digestibility of all amino acids except tryptophan between the brooding and growing phases, aligning with findings in broilers fed wheat bran [6]. However, no significant difference was found in the SID AA between the two phases, which is similar to the findings of Kang et al. [25], who compared amino acid digestibility in laying hens of different ages and reported that most amino acids did not differ significantly across ages. Evidence indicates that the major developmental changes in intestinal morphology, digestive enzyme activity, and nutrient absorptive capacity in chicks occur predominantly during the early post-hatch period, after which digestive function tends to stabilize [32,33]. Thus, although the two experimental phases in the present study were approximately 60 days apart, both the 31-day-old and 95-day-old pullets had already passed the rapid gastrointestinal development phase, and the potential for further age-related improvement in amino acid digestion and absorption capacity was likely limited. This may partly explain the lack of significant differences in SID AA between the two phases. In contrast, Yun et al. [34] reported higher SID AA in 14-d-old broilers compared to 28-d-old broilers, whereas the opposite was reported for corn-fed broilers [35]. These discrepancies across studies may be attributed to differences in poultry species [36], age [6], and feed ingredient sources [16].

By leveraging the correlation between the chemical composition of feed ingredients and SID AA and applying multivariate linear regression to construct predictive equations, the nutritional value of feed ingredients can be estimated, thereby facilitating the precise formulation of diets. The prediction equations provide a practical tool for assessing the nutritional quality of feedstuffs. This conclusion has been validated in soybean meal [16] and wheat [34]. It is noteworthy that, when constructing the equations, we observed that the correlation between SID AA and CP exhibited stage-specific differences: During the brooding phase, the correlation coefficients between CP and the SID of all amino acids except tryptophan were negative, whereas during the growing phase, most of these coefficients shifted to numerically positive values. A similar trend was reported in wheat bran studies [6]. However, none of these correlation coefficients reached statistical significance. Therefore, this numerical shift may partly reflect the small sample size, which could lead to instability in the correlation estimates. Similarly, the possibility that these discrepancies may also result from the influence of poultry breed differences [37] and physiological stage specificity [25] cannot be excluded. We also observed that TS showed positive correlations with SID AA at both the brooding and growing phases. This finding is not directly aligned with the view that the rapid digestion of wheat starch may lead to glucose competing with free amino acids for sodium-dependent co-transport absorption in the small intestine, thereby inhibiting amino acid absorption [38,39]. The apparent discrepancy in our results may stem from the compositional characteristics of WS: their starch content showed a significant negative correlation with CF content. An increase in starch proportion leads to a decrease in fiber content. High fiber can form a physical barrier within the gut, hindering substrate access for digestive enzymes and entrapping nutrients [40]. Therefore, reduced fiber would alleviate this barrier effect, thereby improving amino acid digestibility. This may account for the positive correlation between TS and SID AA observed in the present study.

We also attempted to incorporate amino acids into the equations and achieved good results. Although the amino acid digestibility of WS may be influenced by a variety of factors, the present study indicates that DM, NDF, ADF, CF, EE, ash, GE, NFE, and certain amino acids could serve as predictors for the SID AA of WS.

## CONCLUSION

In summary, the results of this study indicate that WS from different sources demonstrate considerable differences in chemical composition and SID AA values. Correlations were observed between SID AA and the conventional nutrients as well as amino acid contents of WS. Based on these correlations, 18 prediction equations were developed for the brooding phase and 16 for the growing phase using stepwise regression. These results provide reference data for the application of WS in pullet diets.

## Figures and Tables

**Table 1:** Ingredient properties of experimental diet and nitrogen-free diet (as-fed basis)

Ingredient	NFD diet	WS diets
Ingredients (%)		
Wheat shorts	-	92.53
Dextrose	64.00	-
Corn starch	22.16	-
Soybean oil	4.00	1.00
Sodium carboxymethyl cellulose	4.00	2.00
Sodium bicarbonate	1.20	-
Stone powder	1.30	1.30
Calcium hydrogen phosphate (anhydrous)	1.90	1.90
NaCl	-	0.30
KCl	0.40	-
MgO	0.07	-
Choline chloride (50%)	0.25	0.25
Vitamin premix1)	0.02	0.02
Mineral premix2)	0.20	0.20
TiO_2_	0.50	0.50
Nutrient levels (%)3)		
Metabolic energy (MJ/kg)	12.58	12.86
CP	0.26	14.33 (12.83–15.88)
Ca	1.04	0.97 (0.96–1.00)
TP	0.44	1.30 (1.05–1.58)

1) Vitamin premix provided the following per kilogram of diets: Vitamin A 12,000 IU, Vitamin D_3_ 2,500 IU, Vitamin E 20 IU, Vitamin K_3_ 1.3 mg, Vitamin B_1_ 2.2 mg, Vitamin B_2_ 8 mg, Vitamin B_6_ 4 mg, Vitamin B_12_ 0.013 mg, nicotinic acid 40 mg, D-pantothenic acid 10 mg, biotin 0.04 mg, folic acid 1 mg.

2) Mineral premix provided the following per kilogram of diets: Fe 80 mg, Cu 8 mg, Mn 110 mg, Zn 65 mg, I 1.1 mg, Se 0.3 mg.

3) For the nutrient levels, CP, Ca and TP were analyzed values, while ME was calculated according to the corresponding values of each raw material in Tables of Feed Composition and Nutritive Values in of each raw material in Tables of Feed Composition and Nutritive Values in China [41].

NFD, nitrogen-free diet; WS, wheat shorts; TiO_2_, titanium dioxide; CP, crude protein; Ca, calcium; TP, total phosphorus.

**Table 2:** Analyzed crude protein and amino acid contents of experimental diets (dry matter basis)

Items	Diets1)										Mean	Min	Max	CV

	WS1	WS2	WS3	WS4	WS5	WS6	WS7	WS8	WS9	WS10				
CP (%)	14.73	17.02	17.66	15.65	17.97	15.18	18.03	16.71	15.15	14.56	16.27	14.56	18.03	7.96
Essential amino acids (EAA, %)														
Lys	0.60	0.79	0.77	0.62	0.72	0.56	0.79	0.79	0.59	0.52	0.68	0.52	0.79	15.25
Met	0.26	0.28	0.30	0.28	0.33	0.26	0.31	0.30	0.26	0.26	0.28	0.26	0.33	7.80
Trp	0.17	0.18	0.19	0.17	0.19	0.15	0.20	0.18	0.16	0.15	0.17	0.15	0.20	10.55
Thr	0.45	0.53	0.52	0.48	0.57	0.45	0.58	0.56	0.47	0.43	0.50	0.43	0.58	10.13
Arg	0.86	1.03	1.03	0.92	1.07	0.83	1.14	1.09	0.81	0.79	0.96	0.79	1.14	12.75
Leu	0.78	0.99	0.92	0.83	1.02	0.83	1.03	1.00	0.89	0.75	0.90	0.75	1.03	10.90
Ile	0.43	0.51	0.49	0.47	0.56	0.45	0.56	0.54	0.47	0.42	0.49	0.42	0.56	9.81
Phe	0.52	0.66	0.62	0.56	0.67	0.56	0.74	0.68	0.65	0.50	0.62	0.50	0.74	11.98
Val	0.65	0.78	0.73	0.67	0.78	0.66	0.83	0.78	0.65	0.60	0.71	0.60	0.83	10.34
His	0.35	0.43	0.44	0.37	0.45	0.35	0.48	0.44	0.40	0.33	0.41	0.33	0.48	11.95
Total EAA	5.09	6.21	6.03	5.36	6.37	5.12	6.65	6.40	5.32	4.76	5.73	4.76	6.65	11.13
Non-essential amino acids (NEAA, %)														
Asp	0.95	1.17	1.09	0.98	1.14	0.89	1.16	1.12	0.85	0.87	1.02	0.85	1.17	11.76
Ser	0.57	0.67	0.65	0.60	0.73	0.60	0.72	0.69	0.64	0.56	0.64	0.56	0.73	8.82
Glu	2.63	2.94	2.79	2.92	3.59	3.02	3.45	3.18	3.38	2.59	3.05	2.59	3.59	10.68
Gly	0.67	0.94	0.78	0.68	0.81	0.68	0.85	0.85	0.65	0.60	0.75	0.60	0.94	13.97
Ala	0.61	0.83	0.74	0.65	0.77	0.62	0.79	0.78	0.59	0.58	0.70	0.58	0.83	12.97
Pro	0.90	0.99	0.99	0.99	1.26	1.03	1.14	1.10	1.23	0.96	1.06	0.90	1.26	10.65
Cys	0.30	0.33	0.33	0.32	0.35	0.30	0.35	0.33	0.31	0.32	0.32	0.30	0.35	5.93
Tyr	0.28	0.39	0.41	0.33	0.41	0.31	0.36	0.42	0.43	0.32	0.37	0.28	0.43	13.73
Total NEAA	6.92	8.24	7.79	7.46	9.05	7.46	8.82	8.48	8.07	6.79	7.91	6.79	9.05	9.15
TAA	12.00	14.45	13.82	12.82	15.42	12.58	15.48	14.87	13.40	11.55	13.64	11.55	15.48	9.76

1) Sources of WS: WS1 (Shaanxi), WS2 (Shandong), WS3 (Sichuan), WS4 (Hebei), WS5 (Shanxi), WS6 (Sichuan), WS7 (Jiangsu), WS8 (Anhui), WS9 (Henan), and WS10 (Beijing).

WS, wheat shorts; Min, minimum; Max, maximum; CV, coefficient of variation; CP, crude protein; Lys, lysine; Met, methionine; Trp, tryptophan; Thr, threonine; Arg, arginine; Leu, leucine; Ile, isoleucine; Phe, phenylalanine; Val, valine; His, histidine; Asp, aspartic acid; Ser, serine; Glu, glutamic acid; Gly, glycine; Ala, alanine; Pro, proline; Cys, cysteine; Tyr, tyrosine; TAA, total amino acids.

**Table 3:** Analyzed conventional nutritional components and amino acid profiles of wheat shorts (WS) from different origins (dry matter basis)

Items	WS1)										Mean	Min	Max	CV

	WS12)	WS2	WS3	WS4	WS5	WS6	WS7	WS8	WS9	WS10				
DM (%)	90.35	89.15	89.07	87.43	89.33	89.25	88.45	89.32	88.82	89.35	89.05	87.43	90.35	0.80
CP (%)	16.23	18.22	18.85	16.94	19.51	15.71	19.23	18.18	17.10	16.25	17.62	15.71	19.51	7.30
GE (MJ/kg)	20.72	21.17	20.90	20.71	20.91	20.46	20.76	20.89	20.32	20.46	20.73	20.32	21.17	1.18
TS (%)	37.21	37.06	40.05	40.98	46.50	42.65	38.42	40.15	51.58	47.39	42.20	37.06	51.58	10.85
NFE (%)	64.75	65.88	66.92	70.00	70.00	74.20	67.94	69.08	75.69	72.38	69.68	64.75	75.69	4.84
EE (%)	3.44	5.27	4.23	3.81	4.42	3.17	4.33	4.95	2.54	3.78	4.00	2.54	5.27	19.48
Ash (%)	4.91	4.16	4.28	3.82	3.09	3.19	4.04	3.48	2.52	3.07	3.66	2.52	4.91	18.54
CF (%)	10.67	6.47	5.71	5.43	2.98	3.73	4.47	4.31	2.14	4.52	5.04	2.14	10.67	44.28
ADF (%)	14.47	8.50	8.62	7.34	4.54	6.63	7.25	6.55	4.12	7.05	7.51	4.12	14.47	35.96
NDF (%)	43.28	35.32	31.25	29.33	25.02	34.06	27.60	31.72	28.02	38.18	32.38	25.02	43.28	16.08
Ca (%)	0.11	0.12	0.10	0.08	0.09	0.09	0.07	0.07	0.08	0.07	0.09	0.07	0.12	20.95
TP (%)	1.03	1.20	1.34	1.17	1.00	0.82	1.06	0.97	0.70	0.71	1.00	0.70	1.34	19.97
PA (%)	2.40	2.44	2.40	2.17	1.97	1.88	2.26	2.12	1.97	2.03	2.16	1.88	2.44	8.87
Essential amino acids (EAA, %)														
Lys	0.77	0.88	0.82	0.65	0.78	0.59	0.85	0.80	0.55	0.70	0.74	0.55	0.88	14.26
Met	0.26	0.28	0.30	0.31	0.36	0.28	0.32	0.30	0.27	0.25	0.29	0.25	0.36	10.80
Trp	0.23	0.20	0.20	0.19	0.23	0.16	0.22	0.19	0.19	0.19	0.20	0.16	0.23	10.41
Thr	0.54	0.63	0.63	0.49	0.57	0.48	0.62	0.60	0.50	0.56	0.56	0.48	0.63	9.56
Arg	1.05	1.21	1.19	0.97	1.08	0.90	1.23	1.16	0.88	1.06	1.07	0.88	1.23	11.30
Leu	0.96	1.10	1.08	0.88	1.04	0.86	1.11	1.09	0.97	1.01	1.01	0.86	1.11	8.36
Ile	0.51	0.58	0.58	0.49	0.56	0.46	0.58	0.57	0.52	0.54	0.54	0.46	0.58	7.56
Phe	0.67	0.70	0.69	0.59	0.70	0.57	0.76	0.70	0.65	0.66	0.67	0.57	0.76	7.92
Val	0.75	0.87	0.86	0.71	0.79	0.66	0.86	0.84	0.71	0.78	0.78	0.66	0.87	9.03
His	0.44	0.50	0.48	0.40	0.47	0.38	0.50	0.47	0.40	0.44	0.45	0.38	0.50	9.03
Total EAA	6.16	6.93	6.82	5.70	6.59	5.35	7.06	6.72	5.65	6.18	6.32	5.35	7.06	9.02
Non-essential amino acids (NEAA, %)														
Asp	1.13	1.34	1.27	1.03	1.15	0.93	1.24	1.21	0.93	1.15	1.14	0.93	1.34	11.41
Ser	0.68	0.77	0.75	0.63	0.74	0.63	0.83	0.76	0.70	0.73	0.72	0.63	0.83	8.29
Glu	3.08	3.28	3.32	3.10	3.65	3.04	3.65	3.48	3.78	3.44	3.38	3.04	3.78	7.35
Gly	0.83	0.94	0.94	0.72	0.82	0.70	0.94	0.90	0.71	0.80	0.83	0.70	0.94	11.09
Ala	0.76	0.91	0.89	0.68	0.78	0.65	0.86	0.85	0.64	0.77	0.78	0.64	0.91	11.82
Pro	0.97	1.07	1.14	1.02	1.25	1.07	1.24	1.19	1.34	1.18	1.15	0.97	1.34	9.55
Cys	0.31	0.33	0.36	0.38	0.43	0.34	0.38	0.35	0.36	0.32	0.35	0.31	0.43	9.08
Tyr	0.40	0.40	0.42	0.35	0.40	0.36	0.49	0.41	0.39	0.40	0.40	0.35	0.49	8.95
Total NEAA	8.15	9.04	9.08	7.91	9.22	7.74	9.65	9.16	8.85	8.78	8.76	7.74	9.65	6.75
TAA	14.31	15.97	15.90	13.61	15.81	13.08	16.71	15.88	14.50	14.97	15.07	13.08	16.71	7.39

1) Sources of WS: WS1 (Shaanxi), WS2 (Shandong), WS3 (Sichuan), WS4 (Hebei), WS5 (Shanxi), WS6 (Sichuan), WS7 (Jiangsu), WS8 (Anhui), WS9 (Henan), and WS10 (Beijing).

2) According to the specifications for wheat shorts, sample WS1 was classified as an off-specification product and was therefore excluded from subsequent correlation analysis and equation development.

Min, minimum; Max, maximum; CV, coefficient of variation; DM, dry matter; CP, crude protein; GE, gross energy; TS, total starch; NFE, nitrogen-free extract; EE, ether extract; CF, crude fiber; ADF, acid detergent fiber; NDF, neutral detergent fiber; Ca, calcium; TP, total phosphorus; PA, phytic acid; Lys, lysine; Met, methionine; Trp, tryptophan; Thr, threonine; Arg, arginine; Leu, leucine; Ile, isoleucine; Phe, phenylalanine; Val, valine; His, histidine; Asp, aspartic acid; Ser, serine; Glu, glutamic acid; Gly, glycine; Ala, alanine; Pro, proline; Cys, cysteine; Tyr, tyrosine; TAA, total amino acids.

**Table 4:** Ileal endogenous amino acid flow (mg/kg of DM feed intake) in the pullets fed a nitrogen-free diet

Item	Ages	

	31 d	95 d
Essential amino acids (EAA)		
Lys	650.70	1,028.38
Met	264.87	264.98
Trp	147.82	130.04
Thr	756.06	1,013.58
Arg	534.35	864.29
Leu	767.61	1,149.28
Ile	460.62	645.72
Phe	415.09	632.51
Val	585.07	889.28
His	257.90	389.90
Non-essential amino acids (NEAA)		
Asp	954.80	1,385.50
Ser	725.06	940.95
Glu	1,293.90	1,954.73
Gly	535.20	762.05
Ala	512.36	761.38
Pro	635.08	820.51
Cys	324.93	405.08
Tyr	352.64	525.69

DM, dry matter; Lys, lysine; Met, methionine; Trp, tryptophan; Thr, threonine; Arg, arginine; Leu, leucine; Ile, isoleucine; Phe, phenylalanine; Val, valine; His, histidine; Asp, aspartic acid; Ser, serine; Glu, glutamic acid; Gly, glycine; Ala, alanine; Pro, proline; Cys, cysteine; Tyr, tyrosine.

**Table 5:** Standard ileal crude protein and amino acid digestibility of pullets during brooding phase (31 d of age) for wheat shorts from different sources

Items	Diets1)										Mean	Min	Max	SEM	p-value

	WS1	WS2	WS3	WS4	WS5	WS6	WS7	WS8	WS9	WS10					
CP (%)	77.84^d^	74.13^d^	78.15bcd	80.92^bc^	83.54^b^	81.33^bc^	79.08bcd	76.40^cd^	90.16^a^	78.07bcd	79.96	74.13	90.16	0.79	<0.001
Essential amino acids (EAA, %)															
Lys	72.99^b^	74.04^b^	74.82^b^	76.86^b^	80.34^b^	73.65^b^	77.55^b^	78.24^b^	89.13^a^	80.22^b^	77.78	72.99	89.13	0.91	<0.001
Met	75.91^de^	72.79^e^	75.95cde	79.56^cd^	85.97^b^	78.91^cd^	77.56cde	76.76cde	91.36^a^	80.56^c^	79.53	72.79	91.36	0.85	<0.001
Trp	70.11	80.07	82.28	81.25	84.75	80.84	85.27	79.27	84.74	79.17	80.73	70.11	84.75	1.22	0.149
Thr	75.79^bc^	68.56^d^	73.45^cd^	79.27^bc^	80.65^b^	77.12^bc^	75.14^bc^	76.61^bc^	89.96^a^	78.39^bc^	77.49	68.56	89.96	0.95	<0.001
Arg	84.99^d^	83.40^d^	84.00^d^	86.07^cd^	90.27^ab^	85.46^d^	84.51^d^	85.69^d^	92.98^a^	89.19^bc^	86.66	83.40	92.98	0.52	<0.001
Leu	82.37^c^	80.08^c^	79.86^c^	83.84^bc^	87.60^b^	83.92^bc^	82.15^c^	83.35^bc^	94.32^a^	87.69^b^	84.52	79.86	94.32	2.04	<0.001
Ile	82.03bcd	77.70^d^	78.89^cd^	83.83^bc^	86.79^b^	84.27^b^	82.45bcd	83.55^bc^	94.32^a^	87.38^b^	84.12	77.70	94.32	0.78	<0.001
Phe	82.52^c^	80.20^c^	80.48^c^	84.44^bc^	88.03^b^	84.75^bc^	84.08^bc^	84.38^bc^	94.13^a^	87.50^b^	85.05	80.20	94.13	0.69	<0.001
Val	79.97bcde	76.45^e^	77.32^de^	81.78bcd	84.81^b^	81.53bcd	79.38cde	81.16bcde	91.14^a^	84.08^bc^	81.76	76.45	91.14	0.72	<0.001
His	79.28^cd^	77.58^d^	79.44^cd^	82.20^bc^	85.92^b^	81.34^cd^	80.31^cd^	80.96^cd^	90.29^a^	83.20^bc^	82.05	77.58	90.29	0.63	<0.001
Non-essential amino acids (NEAA, %)															
Asp	76.06^bc^	72.68^c^	73.90^c^	78.73^bc^	80.81^b^	75.80^bc^	75.21^bc^	76.80^bc^	87.17^a^	78.55^bc^	77.57	72.68	87.17	0.77	<0.001
Ser	78.94^bc^	72.86^d^	76.54^cd^	82.10^bc^	83.90^b^	80.70^bc^	78.07^cd^	78.89^bc^	91.63^a^	81.69^bc^	80.53	72.86	91.63	0.83	<0.001
Glu	88.95^cd^	85.66^e^	86.73^de^	90.45^bc^	92.58^b^	90.83^bc^	88.97^cd^	89.17^cd^	96.20^a^	91.07^bc^	90.06	85.66	96.20	0.47	<0.001
Gly	74.89^c^	75.52^c^	72.99^c^	77.63^bc^	81.95^b^	77.61^bc^	72.59^c^	76.96^bc^	87.28^a^	77.67^bc^	77.51	72.59	87.28	0.76	<0.001
Ala	77.35^c^	76.29^c^	75.77^c^	79.11^bc^	82.89^b^	78.71^bc^	77.02^c^	79.05^bc^	88.54^a^	80.23^bc^	79.50	75.77	88.54	0.68	<0.001
Pro	87.06^cd^	83.41^d^	85.04^d^	89.86^bc^	91.99^b^	89.81^bc^	85.87^cd^	89.68^bc^	96.39^a^	89.93^bc^	88.90	83.41	96.39	0.63	<0.001
Cys	71.44^de^	64.95^f^	69.20^ef^	78.05^bc^	79.82^b^	75.91bcd	72.61cde	71.39^de^	85.91^a^	71.75^de^	74.10	64.95	85.91	0.97	<0.001
Tyr	80.45^d^	79.37^d^	80.82^d^	82.63^cd^	89.63^b^	83.49^cd^	79.95^d^	83.72^cd^	94.98^a^	87.33^bc^	84.24	79.37	94.98	2.17	<0.001
TAA	80.54^de^	78.68^e^	79.74^de^	83.04bcde	87.10^b^	83.65bcd	81.70cde	82.93bcde	92.69^a^	85.30^bc^	83.54	78.68	92.69	0.67	<0.001

Data represent the means of 6 replicates (n = 9).

1) Sources of WS: WS1 (Shaanxi), WS2 (Shandong), WS3 (Sichuan), WS4 (Hebei), WS5 (Shanxi), WS6 (Sichuan), WS7 (Jiangsu), WS8 (Anhui), WS9 (Henan), and WS10 (Beijing).

a–f Means within a row lacking a common superscript differ (p<0.05).

Min, minimum; Max, maximum; SEM, standard error of the mean; Lys, lysine; Met, methionine; Trp, tryptophan; Thr, threonine; Arg, arginine; Leu, leucine; Ile, isoleucine; Phe, phenylalanine; Val, valine; His, histidine; Asp, aspartic acid; Ser, serine; Glu, glutamic acid; Gly, glycine; Ala, alanine; Pro, proline; Cys, cysteine; Tyr, tyrosine; TAA, total amino acids.

**Table 6:** Standard ileal crude protein and amino acid digestibility of pullets during growing phase (95 days of age) for wheat shorts from different sources

Items	Diets1)										Mean	Min	Max	SEM	p-value

	WS1	WS2	WS3	WS4	WS5	WS6	WS7	WS8	WS9	WS10					
CP (%)	69.94^f^	78.32^cd^	75.38^de^	80.71^bc^	86.89^a^	83.33^ab^	76.72cde	74.13^e^	85.03^a^	77.28cde	78.77	69.94	86.89	0.76	<0.001
Essential amino acids (EAA, %)															
Lys	66.70^e^	84.40^b^	80.94^d^	77.98^cd^	90.82^a^	84.04^b^	77.65^cd^	82.38^bc^	87.59^ab^	72.72^d^	80.52	66.70	90.82	1.11	<0.001
Met	66.89^e^	78.74^bc^	70.92^de^	79.29^bc^	86.44^a^	83.00^ab^	73.82^cd^	75.12^cd^	84.56^ab^	78.77^bc^	77.75	66.89	86.44	0.96	<0.001
Trp	67.70^c^	75.23^bc^	83.30^ab^	84.87^ab^	87.87^a^	78.24^ab^	77.99^ab^	78.76^ab^	83.89^ab^	79.19^ab^	79.70	67.70	87.87	1.15	0.003
Thr	64.10^c^	75.19^b^	67.09^c^	74.16^b^	84.69^a^	80.82^a^	72.59^b^	75.29^b^	85.54^a^	65.31^c^	74.48	64.10	85.54	1.10	<0.001
Arg	82.15^f^	89.35^cd^	85.02^e^	87.93^de^	96.34^a^	91.04^bc^	86.50^de^	88.49^cd^	93.72^ab^	86.99^de^	88.75	82.15	96.34	0.60	<0.001
Leu	77.91^f^	88.18^bc^	79.77^f^	84.60cde	93.79^a^	91.02^ab^	83.67^de^	86.31^cd^	92.84^a^	81.40^ef^	85.95	77.91	93.79	0.79	<0.001
Ile	77.14^g^	86.99^bc^	78.31^fg^	83.72cde	92.60^a^	89.43^ab^	82.00def	84.30^cd^	90.64^ab^	79.60efg	84.47	77.14	92.60	0.80	<0.001
Phe	77.81^g^	87.87^cd^	80.94^f^	84.89^de^	94.25^a^	89.94^bc^	84.79^de^	86.69^d^	92.70^ab^	83.34^ef^	86.32	77.81	94.25	0.72	<0.001
Val	74.62^f^	84.27^bc^	76.42^ef^	80.92^cd^	90.26^a^	86.33^ab^	79.88^de^	82.12^cd^	88.12^ab^	76.38^ef^	81.93	74.62	90.26	0.78	<0.001
His	73.02^e^	82.53^b^	77.14^d^	80.45bcd	90.88^a^	83.40^b^	78.88^cd^	82.20^bc^	87.75^a^	77.00^d^	81.32	73.02	90.88	0.76	<0.001
Non-essential amino acids (NEAA, %)															
Asp	67.51^f^	79.80^bc^	70.85^ef^	76.15^cd^	85.77^a^	81.05abc	74.53^de^	78.21bcd	83.21^ab^	68.61^f^	76.57	67.51	85.77	0.93	<0.001
Ser	69.06^d^	78.14^c^	71.71^d^	78.26^c^	87.80^ab^	84.45^b^	76.83^c^	78.87^c^	88.55^a^	72.46^d^	78.61	69.06	88.55	0.94	<0.001
Glu	85.00^e^	90.12^b^	85.86^de^	90.22^b^	95.41^a^	93.51^a^	88.82^bc^	90.27^b^	95.39^a^	87.56^cd^	90.22	85.00	95.41	0.51	<0.001
Gly	66.94^g^	80.18^bc^	70.78efg	75.00^de^	86.51^a^	79.52^bc^	73.44def	77.46^cd^	83.77^ab^	69.17^fg^	76.28	66.94	86.51	0.92	<0.001
Ala	71.26^d^	83.17^b^	74.34^cd^	77.58^c^	87.96^a^	84.46^ab^	77.35^c^	81.92^b^	84.78^ab^	72.30^d^	79.51	71.26	87.96	0.86	<0.001
Pro	82.32^f^	85.48def	82.74^f^	88.89bcd	92.42^ab^	90.81abc	84.97^ef^	87.74cde	94.20^a^	82.87^f^	87.24	82.32	94.20	0.66	<0.001
Cys	60.36^d^	67.30^cd^	64.36^d^	77.35^ab^	80.72^a^	71.43^bc^	64.40^d^	64.36^d^	81.43^a^	71.58^bc^	70.33	60.36	81.43	1.12	<0.001
Tyr	79.57^e^	89.14^bc^	85.87^cd^	86.38^cd^	94.36^a^	92.01^ab^	83.86^d^	89.16^bc^	94.08^a^	85.88^bc^	88.03	79.57	94.36	0.70	<0.001
TAA	75.70^g^	84.61^cd^	78.38^fg^	83.33^d^	91.32^a^	87.64^bc^	81.79^de^	83.99^d^	90.61^ab^	79.20^ef^	83.66	75.70	91.32	0.75	<0.001

Data represent the means of 6 replicates (n = 6).

1) Sources of WS: WS1 (Shaanxi), WS2 (Shandong), WS3 (Sichuan), WS4 (Hebei), WS5 (Shanxi), WS6 (Sichuan), WS7 (Jiangsu), WS8 (Anhui), WS9 (Henan), and WS10 (Beijing).

a–g Means within a row lacking a common superscript differ (p<0.05).

Min, minimum; Max, maximum; SEM, standard error of the mean; Lys, lysine; Met, methionine; Trp, tryptophan; Thr, threonine; Arg, arginine; Leu, leucine; Ile, isoleucine; Phe, phenylalanine; Val, valine; His, histidine; Asp, aspartic acid; Ser, serine; Glu, glutamic acid; Gly, glycine; Ala, alanine; Pro, proline; Cys, cysteine; Tyr, tyrosine; TAA, total amino acids.

**Table 7:** Comparison of SID AA of wheat shorts fed to pullets between brooding (31 d) and growing (95 d) phases

Items	SID		SEM	p-value

	31 d	95 d		
Essential amino acids (EAA, %)				
Lys	77.78	80.52	1.35	0.325
Met	79.53	77.75	1.28	0.502
Trp	80.73	79.70	1.12	0.644
Thr	77.49	74.48	1.49	0.323
Arg	86.66	88.75	0.83	0.217
Leu	84.52	85.95	1.09	0.526
Ile	84.12	84.47	1.10	0.877
Phe	85.05	86.32	1.02	0.546
Val	81.76	81.93	1.04	0.938
His	82.05	81.32	1.00	0.726
Non-essential amino acids (NEAA, %)				
Asp	77.57	76.57	1.15	0.675
Ser	80.53	78.61	1.30	0.475
Glu	90.06	90.22	0.73	0.918
Gly	77.51	76.28	1.20	0.620
Ala	79.50	79.51	1.06	0.994
Pro	88.90	87.24	0.89	0.367
Cys	74.10	70.33	1.53	0.227
Tyr	84.24	88.03	1.14	0.097

SID AA, standardized ileal digestibility of amino acids; SEM, standard error of the mean; Lys, lysine; Met, methionine; Trp, tryptophan; Thr, threonine; Arg, arginine; Leu, leucine; Ile, isoleucine; Phe, phenylalanine; Val, valine; His, histidine; Asp, aspartic acid; Ser, serine; Glu, glutamic acid; Gly, glycine; Ala, alanine; Pro, proline; Cys, cysteine; Tyr, tyrosine.

**Table 8:** Prediction equations for standardized ileal digestible amino acids (SID AA) in pullets fed wheat shorts during brooding and growing phases

Amino acids	Basis	Prediction equation	R^2^	p-value
Brooding phase				
Met	TS, NDF	SID Met = 51.929+1.015TS−0.494NDF	0.976	<0.001
Phe	TS, ADF	SID Phe = 72.281+0.499TS−1.232ADF	0.909	<0.001
Lys	TS, Glu	SID Lys = 21.992+0.611TS+8.846Glu	0.843	0.002
His	TS, NDF	SID His = 61.093+0.704TS−0.284NDF	0.961	<0.001
Arg	ADF	SID Arg = 99.383−1.863ADF	0.757	0.001
Thr	ADF, EE	SID Thr = 106.468−2.370ADF−3.163EE	0.877	<0.001
Val	ADF, GE	SID Val = 223.787−2.034ADF−6.180GE	0.898	<0.001
Ile	ADF, GE	SID Ile = 271.430−2.069ADF−8.352GE	0.922	<0.001
Leu	NFE	SID Leu = 4.687+1.140NFE	0.641	0.006
Trp	NDF	SID Trp = 97.339−0.493NDF	0.681	0.004
Asp	TS	SID Asp = 42.588+0.822TS	0.785	<0.001
Ser	TS, DM, CF	SID Ser = 311.171+0.684TS−2.837DM−1.698CF	0.975	<0.001
Glu	ADF, GE	SID Glu = 196.758−1.448ADF−4.671GE	0.962	<0.001
Gly	ADF	SID Gly = 95.300−2.599ADF	0.760	0.001
Ala	TS	SID Ala = 47.547+0.753TS	0.803	<0.001
Pro	DM, Ash	SID Pro = 273.900−6.622Ash−1.817DM	0.918	<0.001
Cys	ADF, DM, EE	SID Cys = 378.671−3.039ADF−3.085DM−2.252EE	0.973	<0.001
Tyr	TS, ADF	SID Tyr = 60.175+0.748TS−1.112ADF	0.970	<0.001
Growing phase				
Met	ADF, Pro	SID Met = 144.950−4.102ADF−32.884Pro	0.748	0.007
Phe	ADF, GE	SID Phe = −4.532−2.804ADF+5.338GE	0.679	0.014
His	ADF, GE, NFE	SID His = −511.489−2.095ADF+23.722GE+1.652NFE	0.900	0.002
Arg	ADF, GE	SID Arg = 8.909−2.404ADF+4.668GE	0.742	0.007
Thr	TS, ADF	SID Thr = 154.539−5.899ADF−0.917TS	0.673	0.015
Val	ADF, GE, His	SID Val = −187.431−2.971ADF+15.307GE−60.471His	0.659	0.039
Ile	ADF, GE, Pro	SID Ile = 2.670−4.211ADF+6.855GE−26.687Pro	0.642	0.044
Leu	ADF, GE, Pro	SID Leu = 14.978−4.089ADF+6.100GE−23.199Pro	0.628	0.049
Trp	Cys, TS	SID Trp = 33.266+87.831Cys+0.376TS	0.689	0.013
Asp	ADF, TS, Pro	SID Asp = 180.026−5.747ADF−0.906TS−21.446Pro	0.623	0.050
Ser	ADF	SID Ser = 102.134−3.336ADF	0.677	0.004
Glu	ADF, CP	SID Glu = 106.550−1.837ADF−0.190CP	0.641	0.020
Gly	ADF, GE	SID Gly = −139.223−3.677ADF+11.639GE	0.630	0.021
Pro	ADF, Pro	SID Pro = 135.008−3.274ADF−21.574Pro	0.815	0.003
Cys	Gly, Trp	SID Cys = 89.524−76.046Gly+228.947Trp	0.868	<0.001
Tyr	Ash, GE	SID Tyr = −84.420−7.728Ash+9.675GE	0.612	0.025

Met, methionine; TS, total starch; NDF, neutral detergent fiber; Phe, phenylalanine; ADF, acid detergent fiber; Lys, lysine; Glu, glutamic acid; His, histidine; Arg, arginine; Thr, threonine; EE, ether extract; Val, valine; GE, gross energy; Ile, isoleucine; Leu, leucine; NFE, nitrogen-free extract; Trp, tryptophan; Asp, aspartic acid; Ser, serine; DM, dry matter; CF, crude fiber; Gly, glycine; Ala, alanine; Pro, proline; Cys, cysteine; Tyr, tyrosine; CP, crude protein.

## SUPPLEMENTARY MATERIAL

**Supplement 1.** Pearson correlation coefficients between conventional nutrient components of wheat shorts and SID AA of wheat shorts in pullets during the brooding phase.

**Supplement 2.** Pearson correlation coefficients between amino acid contents of wheat shorts and SID AA of wheat shorts in pullets during the brooding phase.

**Supplement 3.** Pearson correlation coefficients between conventional nutrient components of wheat shorts and SID AA of wheat shorts in pullets during the growing phase.

**Supplement 4.** Pearson correlation coefficients between amino acid contents of wheat shorts and SID AA of wheat shorts in pullets during the growing phase.
