# Supplementary material for: Determination and prediction of standardized ileal amino acid digestibility of wheat shorts for pullets during the brooding and growing phases
Source: Anim Biosci. 2026 Jun 15;39(9):260107. doi: 10.5713/ab.260107 (PMC13547626; doi:10.5713/ab.260107)
Supplement: Supplementary file 1 [file ab-260107-Supplementary-1.pdf]

Supplement 1. Pearson correlation coefficients between conventional nutrient components of wheat shorts and SID AA of wheat shorts in pullets during the brooding phase

Items	DM	CP	GE	TS	NFE	EE	Ash	CF	ADF	NDF	Ca	TP	PA
Essential amino acids													
SID Lys	0.001	-0.052	-0.561	0.842**	0.613	-0.554	-0.762*	-0.759*	-0.803**	-0.375	-0.440	-0.628	-0.469
SID Met	-0.020	-0.133	-0.644	0.922**	0.757*	-0.716*	-0.852**	-0.888**	-0.921**	-0.504	-0.356	-0.634	-0.700*
SID Trp	-0.219	0.514	-0.128	0.281	0.109	-0.305	-0.147	-0.518	-0.505	-0.849**	-0.095	0.016	-0.105
SID Thr	-0.145	-0.274	-0.751*	0.887**	0.821**	-0.788*	-0.847**	-0.862**	-0.869**	-0.444	-0.510	-0.666	-0.718*
SID Arg	0.118	-0.193	-0.621	0.968**	0.743*	-0.621	-0.903**	-0.832**	-0.888**	-0.309	-0.376	-0.722*	-0.699*
SID Leu	0.062	-0.313	-0.723*	0.956**	0.828**	-0.711*	-0.930**	-0.852**	-0.881**	-0.258	-0.421	-0.799**	-0.728*
SID Ile	0.011	-0.350	-0.790*	0.934**	0.869**	-0.755*	-0.933**	-0.885**	-0.883**	-0.282	-0.542	-0.826**	-0.783*
SID Phe	0.028	-0.272	-0.738*	0.929**	0.829**	-0.716*	-0.922**	-0.895**	-0.907**	-0.333	-0.498	-0.803**	-0.750*
SID Val	0.010	-0.316	-0.738*	0.944**	0.846**	-0.734*	-0.931**	-0.878**	-0.902**	-0.319	-0.469	-0.780*	-0.764*
SID His	-0.011	-0.173	-0.658	0.936**	0.772*	-0.707*	-0.876**	-0.880**	-0.918**	-0.453	-0.392	-0.667*	-0.702*
Non-essential amino acids													
SID Asp	-0.109	-0.204	-0.646	0.901**	0.752*	-0.688*	-0.849**	-0.825**	-0.886**	-0.435	-0.416	-0.645	-0.653
SID Ser	-0.127	-0.302	-0.759*	0.909**	0.842**	-0.803**	-0.868**	-0.866**	-0.878**	-0.423	-0.471	-0.685*	-0.745*
SID Glu	-0.052	-0.320	-0.767*	0.912**	0.872**	-0.778*	-0.918**	-0.914**	-0.920**	-0.397	-0.495	-0.766*	-0.826**
SID Gly	0.077	-0.259	-0.523	0.868**	0.744*	-0.601	-0.881**	-0.791*	-0.889**	-0.320	-0.143	-0.645	-0.674*
SID Ala	0.042	-0.203	-0.611	0.910**	0.766*	-0.655	-0.894**	-0.859**	-0.920**	-0.386	-0.317	-0.696*	-0.678*
SID Pro	-0.013	-0.348	-0.713*	0.893**	0.857**	-0.717*	-0.927**	-0.885**	-0.913**	-0.355	-0.488	-0.740*	-0.833**
SID Cys	-0.265	-0.210	-0.665	0.781*	0.770*	-0.768*	-0.766*	-0.849**	-0.875**	-0.601	-0.405	-0.540	-0.731*
SID Tyr	0.188	-0.219	-0.624	0.969**	0.774*	-0.642	-0.925**	-0.859**	-0.901**	-0.293	-0.323	-0.727*	-0.716*

*$0.01 < p \leq 0.05$; **$0.001 < p \leq 0.01$.

Abbreviations: ADF, acid detergent fiber; Ala, alanine; Arg, arginine; Asp, aspartic acid; Ca, calcium; CV, coefficient of variation; CF, crude fiber; CP, crude protein; Cys, cysteine; DM, dry matter; EE, ether extract; GE, gross energy; Glu, glutamic acid; Gly, glycine; His, histidine; Ile, isoleucine; Leu, leucine; Lys, lysine; Met, methionine; Max, maximum; Min, minimum; NDF, neutral detergent fiber; NFE, nitrogen-free extract; PA, phytic acid; Phe, phenylalanine; Pro, proline; Ser, serine; Thr, threonine; TP, total phosphorus; Trp, tryptophan; TS, total starch; Tyr, tyrosine; Val, valine.
