# Supplementary material for: Determination and prediction of standardized ileal amino acid digestibility of wheat shorts for pullets during the brooding and growing phases
Source: Anim Biosci. 2026 Jun 15;39(9):260107. doi: 10.5713/ab.260107 (PMC13547626; doi:10.5713/ab.260107)
Supplement: Supplementary file 2 [file ab-260107-Supplementary-2.pdf]

Supplement 2. Pearson correlation coefficients between amino acid contents of wheat shorts and SID AA of wheat shorts in pullets during the brooding phase

Items	Lys	Met	Trp	Thr	Arg	Leu	Ile	Phe	Val	His	Asp	Ser	Glu	Gly	Ala	Pro	Cys	Tyr
Essential amino acids																		
SID Lys	-0.489	-0.123	0.159	-0.322	-0.447	-0.055	-0.044	0.062	-0.273	-0.276	-0.446	-0.005	0.769*	-0.396	-0.453	0.819**	0.195	0.008
SID Met	-0.669*	0.070	0.100	-0.572	-0.677*	-0.352	-0.327	-0.197	-0.554	-0.501	-0.681*	-0.289	0.587	-0.643	-0.688*	0.695*	0.412	-0.212
SID Trp	-0.048	0.553	0.590	-0.016	-0.058	0.110	0.135	0.341	-0.016	0.097	-0.163	0.245	0.624	-0.025	-0.138	0.626	0.728*	0.426
SID Thr	-0.774*	-0.045	-0.080	-0.669*	-0.749*	-0.451	-0.435	-0.309	-0.635	-0.626	-0.775*	-0.388	0.496	-0.715*	-0.770*	0.626	0.297	-0.271
SID Arg	-0.614	-0.065	0.088	-0.502	-0.619	-0.274	-0.250	-0.161	-0.480	-0.442	-0.585	-0.235	0.604	-0.598	-0.611	0.692*	0.265	-0.237
SID Leu	-0.708*	-0.184	-0.048	-0.586	-0.696*	-0.348	-0.347	-0.230	-0.561	-0.535	-0.678*	-0.285	0.568	-0.665	-0.694*	0.675*	0.153	-0.251
SID Ile	-0.751*	-0.169	-0.100	-0.635	-0.724*	-0.400	-0.408	-0.262	-0.609	-0.588	-0.737*	-0.315	0.541	-0.699*	-0.741*	0.667*	0.162	-0.229
SID Phe	-0.698*	-0.120	-0.018	-0.583	-0.679*	-0.336	-0.344	-0.189	-0.557	-0.522	-0.691*	-0.248	0.605	-0.651	-0.697*	0.715*	0.210	-0.177
SID Val	-0.745*	-0.128	-0.074	-0.636	-0.733*	-0.401	-0.398	-0.275	-0.608	-0.584	-0.730*	-0.340	0.537	-0.704*	-0.739*	0.655	0.207	-0.282
SID His	-0.683*	0.007	0.057	-0.576	-0.683*	-0.346	-0.324	-0.208	-0.550	-0.514	-0.681*	-0.295	0.589	-0.648	-0.688*	0.696*	0.350	-0.237
Non-essential amino acids																		
SID Asp	-0.694*	-0.036	0.023	-0.594	-0.688*	-0.358	-0.332	-0.230	-0.549	-0.532	-0.680*	-0.317	0.565	-0.657	-0.696*	0.653	0.307	-0.274
SID Ser	-0.793*	-0.050	-0.085	-0.697*	-0.778*	-0.486	-0.469	-0.342	-0.668*	-0.648	-0.794*	-0.420	0.472	-0.749*	-0.796*	0.603	0.298	-0.308
SID Glu	-0.782*	-0.031	-0.080	-0.707*	-0.778*	-0.489	-0.489	-0.329	-0.687*	-0.640	-0.802**	-0.400	0.479	-0.761*	-0.805**	0.613	0.299	-0.288
SID Gly	-0.683*	-0.074	-0.077	-0.608	-0.728*	-0.392	-0.379	-0.322	-0.585	-0.547	-0.670*	-0.400	0.440	-0.680*	-0.686*	0.534	0.226	-0.452
SID Ala	-0.670*	-0.070	0.002	-0.560	-0.679*	-0.315	-0.307	-0.204	-0.531	-0.499	-0.659	-0.281	0.587	-0.632	-0.666	0.683*	0.255	-0.275
SID Pro	-0.789*	-0.044	-0.160	-0.717*	-0.791*	-0.502	-0.498	-0.390	-0.689*	-0.670*	-0.799**	-0.467	0.413	-0.768*	-0.796*	0.551	0.262	-0.404
SID Cys	-0.768*	0.170	-0.029	-0.742*	-0.783*	-0.558	-0.535	-0.379	-0.714*	-0.659	-0.826**	-0.479	0.381	-0.767*	-0.825**	0.503	0.489	-0.327
SID Tyr	-0.648	-0.099	0.002	-0.517	-0.654	-0.289	-0.275	-0.203	-0.504	-0.478	-0.618	-0.273	0.576	-0.609	-0.625	0.690*	0.221	-0.284

*0.01< p ≤0.05; **0.001< p ≤0.01.

Abbreviation: Ala, alanine; Arg, arginine; Asp, aspartic acid; Cys, cysteine; Glu, glutamic acid; Gly, glycine; His, histidine; Ile, isoleucine; Lys, lysine; Leu, leucine; Met, methionine; Phe, phenylalanine; Pro, proline; Ser, serine; Thr, threonine; Trp, tryptophan; Tyr, tyrosine; Val, valine.
