# Supplementary material for: Determination and prediction of standardized ileal amino acid digestibility of wheat shorts for pullets during the brooding and growing phases
Source: Anim Biosci. 2026 Jun 15;39(9):260107. doi: 10.5713/ab.260107 (PMC13547626; doi:10.5713/ab.260107)
Supplement: Supplementary file 3 [file ab-260107-Supplementary-3.pdf]

Supplement 3. Pearson correlation coefficients between conventional nutrient components of wheat shorts and SID AA of wheat shorts in pullets during the growing phase

Items	DM	CP	GE	TS	NFE	EE	Ash	CF	ADF	NDF	Ca	TP	PA
Essential amino acids													
SID Lys	0.302	0.315	0.178	0.255	0.139	-0.044	-0.329	-0.480	-0.583	-0.535	0.427	-0.019	-0.250
SID Met	0.113	-0.308	-0.378	0.670*	0.654	-0.458	-0.767*	-0.661	-0.772*	-0.230	0.054	-0.578	-0.736*
SID Trp	-0.262	0.223	-0.155	0.524	0.236	-0.336	-0.327	-0.442	-0.545	-0.684*	-0.137	0.045	-0.321
SID Thr	0.059	0.018	-0.182	0.428	0.475	-0.341	-0.584	-0.704*	-0.789*	-0.567	0.097	-0.345	-0.557
SID Arg	0.244	0.034	-0.162	0.594	0.481	-0.279	-0.693*	-0.723*	-0.846**	-0.466	0.111	-0.431	-0.628
SID Leu	0.206	-0.043	-0.149	0.442	0.473	-0.272	-0.620	-0.665	-0.762*	-0.407	0.173	-0.414	-0.581
SID Ile	0.169	-0.027	-0.104	0.404	0.432	-0.241	-0.576	-0.619	-0.731*	-0.418	0.215	-0.359	-0.553
SID Phe	0.236	0.011	-0.163	0.513	0.481	-0.268	-0.668*	-0.718*	-0.823**	-0.445	0.109	-0.454	-0.608
SID Val	0.171	0.051	-0.061	0.381	0.388	-0.210	-0.545	-0.623	-0.735*	-0.478	0.220	-0.311	-0.516
SID His	0.199	0.162	-0.041	0.486	0.368	-0.185	-0.594	-0.678*	-0.815**	-0.564	0.150	-0.304	-0.520
Non-essential amino acids													
SID Asp	0.120	0.127	0.042	0.271	0.287	-0.119	-0.447	-0.553	-0.679*	-0.525	0.247	-0.200	-0.425
SID Ser	0.082	-0.050	-0.281	0.526	0.567	-0.416	-0.675*	-0.772*	-0.847**	-0.539	0.021	-0.446	-0.650
SID Glu	0.096	-0.111	-0.299	0.554	0.594	-0.413	-0.713*	-0.764*	-0.851**	-0.476	0.011	-0.494	-0.691*
SID Gly	0.184	0.162	0.057	0.341	0.280	-0.106	-0.482	-0.564	-0.706*	-0.508	0.280	-0.218	-0.407
SID Ala	0.245	0.130	0.091	0.218	0.250	-0.048	-0.423	-0.522	-0.631	-0.440	0.285	-0.204	-0.411
SID Pro	-0.072	-0.148	-0.364	0.560	0.630	-0.506	-0.693*	-0.765*	-0.847**	-0.556	-0.071	-0.437	-0.682*
SID Cys	-0.214	-0.245	-0.457	0.771*	0.638	-0.589	-0.713*	-0.628	-0.765*	-0.409	-0.080	-0.442	-0.628
SID Tyr	0.381	-0.101	-0.196	0.579	0.534	-0.331	-0.699*	-0.686*	-0.769*	-0.303	0.215	-0.436	-0.613

*0.01< p ≤0.05; **0.001< p ≤0.01.

Abbreviations: ADF, acid detergent fiber; Ala, alanine; Arg, arginine; Asp, aspartic acid; Ca, calcium; CV, coefficient of variation; CF, crude fiber; CP, crude protein; Cys, cysteine; DM, dry matter; EE, ether extract; GE, gross energy; Glu, glutamic acid; Gly, glycine; His, histidine; Ile, isoleucine; Leu, leucine; Lys, lysine; Met, methionine; Max, maximum; Min, minimum; NDF, neutral detergent fiber; NFE, nitrogen-free extract; PA, phytic acid; Phe, phenylalanine; Pro, proline; Ser, serine; Thr, threonine; TP, total phosphorus; Trp, tryptophan; TS, total starch; Tyr, tyrosine; Val, valine.
