# Supplementary material for: Determination and prediction of standardized ileal amino acid digestibility of wheat shorts for pullets during the brooding and growing phases
Source: Anim Biosci. 2026 Jun 15;39(9):260107. doi: 10.5713/ab.260107 (PMC13547626; doi:10.5713/ab.260107)
Supplement: Supplementary file 4 [file ab-260107-Supplementary-4.pdf]

Supplement 4. Pearson correlation coefficients between amino acid contents of wheat shorts and SID AA of wheat shorts in pullets during the growing phase

Items	Lys	Met	Trp	Thr	Arg	Leu	Ile	Phe	Val	His	Asp	Ser	Glu	Gly	Ala	Pro	Cys	Tyr
Essential amino acids																		
SID Lys	-0.093	0.434	0.195	-0.106	-0.218	-0.002	-0.004	0.013	-0.136	-0.036	-0.191	-0.080	0.283	-0.134	-0.148	0.324	0.486	-0.197
SID Met	-0.592	0.087	-0.072	-0.659	-0.708*	-0.532	-0.531	-0.437	-0.669*	-0.542	-0.642	-0.499	0.146	-0.719*	-0.687*	0.220	0.296	-0.548
SID Trp	-0.317	0.540	0.352	-0.335	-0.365	-0.272	-0.154	-0.171	-0.309	-0.270	-0.357	-0.320	0.258	-0.383	-0.384	0.290	0.761*	-0.275
SID Thr	-0.428	0.327	0.023	-0.476	-0.534	-0.316	-0.344	-0.207	-0.480	-0.364	-0.549	-0.295	0.289	-0.484	-0.523	0.361	0.467	-0.302
SID Arg	-0.375	0.328	0.158	-0.418	-0.500	-0.254	-0.257	-0.161	-0.430	-0.294	-0.463	-0.255	0.362	-0.477	-0.472	0.416	0.492	-0.340
SID Leu	-0.389	0.245	0.000	-0.444	-0.511	-0.293	-0.330	-0.211	-0.460	-0.330	-0.493	-0.280	0.251	-0.470	-0.481	0.315	0.363	-0.344
SID Ile	-0.366	0.286	0.021	-0.443	-0.499	-0.309	-0.337	-0.227	-0.458	-0.322	-0.475	-0.300	0.205	-0.467	-0.471	0.257	0.396	-0.369
SID Phe	-0.366	0.280	0.096	-0.410	-0.484	-0.237	-0.267	-0.138	-0.422	-0.285	-0.467	-0.215	0.357	-0.451	-0.462	0.411	0.418	-0.284
SID Val	-0.320	0.342	0.075	-0.392	-0.452	-0.252	-0.278	-0.167	-0.405	-0.270	-0.436	-0.248	0.249	-0.414	-0.426	0.296	0.445	-0.316
SID His	-0.282	0.419	0.216	-0.330	-0.409	-0.163	-0.164	-0.076	-0.333	-0.205	-0.386	-0.183	0.389	-0.374	-0.382	0.422	0.552	-0.280
Non-essential amino acids																		
SID Asp	-0.245	0.404	0.097	-0.330	-0.378	-0.198	-0.222	-0.125	-0.335	-0.209	-0.371	-0.213	0.225	-0.337	-0.353	0.253	0.472	-0.293
SID Ser	-0.500	0.286	0.001	-0.539	-0.600	-0.368	-0.396	-0.247	-0.545	-0.426	-0.614	-0.332	0.313	-0.558	-0.593	0.401	0.456	-0.325
SID Glu	-0.523	0.251	-0.023	-0.574	-0.629	-0.406	-0.432	-0.289	-0.579	-0.456	-0.628	-0.370	0.280	-0.600	-0.621	0.364	0.422	-0.381
SID Gly	-0.218	0.379	0.160	-0.280	-0.353	-0.135	-0.149	-0.071	-0.285	-0.160	-0.324	-0.163	0.301	-0.307	-0.315	0.320	0.467	-0.278
SID Ala	-0.181	0.372	0.065	-0.259	-0.315	-0.139	-0.178	-0.091	-0.278	-0.153	-0.307	-0.164	0.198	-0.267	-0.278	0.233	0.400	-0.265
SID Pro	-0.624	0.258	-0.097	-0.667*	-0.711*	-0.503	-0.513	-0.387	-0.656	-0.567	-0.725*	-0.483	0.233	-0.676*	-0.709*	0.334	0.456	-0.452
SID Cys	-0.671*	0.141	0.043	-0.709*	-0.751*	-0.577	-0.513	-0.456	-0.680*	-0.599	-0.685*	-0.558	0.218	-0.772*	-0.752*	0.271	0.445	-0.558
SID Tyr	-0.452	0.135	-0.095	-0.439	-0.563	-0.299	-0.317	-0.286	-0.471	-0.385	-0.507	-0.355	0.234	-0.493	-0.483	0.344	0.277	-0.462

*0.01< p ≤0.05; **0.001< p ≤0.01.

Abbreviation: Ala, alanine; Arg, arginine; Asp, aspartic acid; Cys, cysteine; Glu, glutamic acid; Gly, glycine; His, histidine; Ile, isoleucine; Lys, lysine; Leu, leucine; Met, methionine; Phe, phenylalanine; Pro, proline; Ser, serine; Thr, threonine; Trp, tryptophan; Tyr, tyrosine; Val, valine.
